# Geocoding processes in cohort studies: methods applied in the EpiFloripa Aging

**DOI:** 10.11606/s1518-8787.2023057004976

**Published:** 2023-11-08

**Authors:** Catharina Cavasin Salvador, Adalberto Aparecido dos Santos Lopes, Danilo Resendes, Fernanda Faccio Demarco, Marcelo Dutra Della Justina, Renato Tibiriçá de Saboya, Cassiano Ricardo Rech, Eleonora d’Orsi

**Affiliations:** I Universidade Estadual de Londrina Programa Associado de Pós-Graduação em Arquitetura e Urbanismo Londrina PR Brasil Universidade Estadual de Londrina. Programa Associado de Pós-Graduação em Arquitetura e Urbanismo. Londrina, PR, Brasil; II Universidade Federal de Santa Catarina Programa de Pós-Graduação em Arquitetura e Urbanismo Florianópolis SC Brasil Universidade Federal de Santa Catarina. Programa de Pós-Graduação em Arquitetura e Urbanismo. Florianópolis, SC, Brasil; III Universidade Federal de Minas Gerais Observatório de Saúde Urbana Belo Horizonte MG Brasil Universidade Federal de Minas Gerais. Observatório de Saúde Urbana. Belo Horizonte, MG, Brasil; IV Universidade Federal de Santa Catarina Programa de Pós-Graduação em Educação Física Florianópolis SC Brasil Universidade Federal de Santa Catarina. Programa de Pós-Graduação em Educação Física. Florianópolis, SC, Brasil; V Universidade Federal de Santa Catarina Centro Tecnológico Departamento de Arquitetura e Urbanismo Florianópolis SC Brasil Universidade Federal de Santa Catarina. Centro Tecnológico. Departamento de Arquitetura e Urbanismo. Florianópolis, SC, Brasil; VI Universidade Federal de Santa Catarina Centro de Desportos Departamento de Educação Física Florianópolis SC Brasil Universidade Federal de Santa Catarina. Centro de Desportos. Departamento de Educação Física. Florianópolis, SC, Brasil; VII Universidade Federal de Santa Catarina Centro de Ciências da Saúde Departamento de Saúde Pública Florianópolis SC Brasil Universidade Federal de Santa Catarina. Centro de Ciências da Saúde. Departamento de Saúde Pública. Florianópolis, SC, Brasil

**Keywords:** Health of Aged Persons, Environment and Public Health, Health Surveys, Geographic Mapping, Geographic Information Systems, Spatial analysis

## Abstract

**OBJECTIVE:**

To describe the process and epidemiological implications of georeferencing in EpiFloripa Aging samples (2009–2019).

**METHOD:**

The EpiFloripa Aging Cohort Study sought to investigate and monitor the living and health conditions of the older adult population (≥ 60) of Florianópolis in three study waves (2009/2010, 2013/2014, 2017/2019). With an automatic geocoding tool, the residential addresses were spatialized, allowing to investigate the effect of the georeferencing sample losses regarding 19 variables, evaluated in the three waves. The influence of different neighborhood definitions (census tracts, Euclidean buffers, and buffers across the street network) was examined in the results of seven variables: area, income, residential density, mixed land use, connectivity, health unit count, and public open space count. Pearson’s correlation coefficients were calculated to evaluate the differences between neighborhood definitions according to three variables: contextual income, residential density, and land use diversity.

**RESULT:**

The losses imposed by geocoding (6%, n = 240) caused no statistically significant difference between the total sample and the geocoded sample. The analysis of the study variables suggests that the geocoding process may have included a higher proportion of participants with better income, education, and living conditions. The correlation coefficients showed little correspondence between measures calculated by the three neighborhood definitions (r = 0.37–0.54). The statistical difference between the variables calculated by buffers and census tracts highlights limitations in their use in the description of geospatial attributes.

**CONCLUSION:**

Despite the challenges related to geocoding, such as inconsistencies in addresses, adequate correction and verification mechanisms provided a high rate of assignment of geographic coordinates, the findings suggest that adopting buffers, favored by geocoding, represents a potential for spatial epidemiological analyses by improving the representation of environmental attributes and the understanding of health outcomes.

## INTRODUCTION

With the increase in the world urban population, a growing number of investigations seek to understand the relationships between urbanized environments and health outcomes^1^. Planning and managing cities efficiently may promote health and well-being, as well as reduce the incidence of chronic non-communicable diseases^2,3^, with a lasting effect^4^. Geographic Information Systems (GIS) are a set of technologies that allow the integration, in the same environment, of variables about different aspects of reality and at different aggregation scales^5,6^. Geographic models based on GIS support in the analysis of health disparities concepts such as neighborhood context, health services availability, physical activity practice, and daily destination accessibility^7^, capable of contributing to work on health and quality of life in cities.

Advances in GIS in the last two decades have increased the specificity with which an individual’s neighborhood environment can be spatially defined^8^. The GIS analyses in the Collective Health field are generally based on the residential location of an individual, which can be defined at various levels of geographic resolution, such as: a) administrative boundaries (neighborhoods, municipalities, or other regionalizations); b) census tracts (territorial unit defined at each census by the Brazilian Institute of Geography and Statistics, IBGE, to control the collection of population data); and, c) latitude and longitude of a residential address. For administrative limits and census tracts, converting the address into a coordinate is unnecessary; however, the correspondence of the address with the territorial limit under study should be observed. On the other hand, the latter requires a process of converting textual addresses into geographic coordinates, known as geocoding^6,11^.

The importance of geocoding for analyzing health data has been evidenced by national surveys^12^.

Geocoding allows the adoption of buffers, a zone around an individual’s home address (point) that establishes a boundary area, defined by a specified maximum distance, where spatial data of interest is aggregated. Buffers define and characterize the neighborhood accurately, helping to manage census tract limitations and the modifiable area unit problem^7^. Despite the importance of the scale to aggregate the environment variables, few studies have examined the influence of different neighborhood definitions in the results of analyses^13^. Thus, the results of the objective attributes of the urban environment acquired with each type of geographical resolution may be different, overestimating or underestimating the real exposure that the participants of an epidemiological study have to the attributes of interest of the investigation.

Although the agility in the spatialization of a large volume of sites is an advantage of geocoding, the conversion process increases the risk of position and classification errors. Previous works have reported variable geocoding rates and losses caused by problematic addresses and poor record quality^14^. Errors can lead to incorrect descriptions of the built environment variables, distorted conclusions about the association between dependent and independent variables, and inadequate public health decisions^11^. International studies use ArcGIS(r)/ArcView(r), a software licensed for geocoding^6^, but point out risks of incorrect localization^6^ and errors when applied in other countries^19^. Other studies hire commercial companies with trained professionals, their own software, and continuous spatial corrections^18^. Therefore, to minimize internal geocoding expenses, high-quality locational data is critical.

The EpiFloripa Aging Cohort Study, conducted in Florianópolis, Santa Catarina, sought to investigate and monitor the living and health conditions of the older population (60 years or older) living in the urban area of the municipality^20^. Publications from this project have, so far, used the census tracts as a spatial unit of analysis and representation of the participants’ neighborhoods^21,22^. With households geocoding, new studies can be developed, applying more specific units of analysis to the urban environment that can effectively be accessed within a certain time interval. However, this process imposes several technological and operational challenges that need to be addressed to ensure reliability and accuracy of the results.

Thus, this study describes the process and epidemiological implications of geocoding the residences of the EpiFloripa Aging Cohort Study (2009–2019) participants. For the latter, more specifically, we: a) compare sociodemographic data, environment and health condition perception obtained for the total sample and the proportion that was geocoded, searching possible distortions; and b) compare the performance of three possible neighborhood definitions from geocoding (census tracts, Euclidean buffers, and buffers across the street network) for some relevant variables, such as income, residential density, mixed land use, and connectivity.

## METHODS

The EpiFloripa Ageing project is a population-based cohort study developed by the Federal University of Santa Catarina^23^. The spatial context of the study involves the entire city of Florianópolis (SC), with 421,240 inhabitants and 11.4% of the population over 60 years of age^19^. The sample selection process was carried out by clusters, in which the first stage units were the census tracts and those of the second stage were the households themselves. Initially, in 2009, the 420 urban census tracts of the municipality were organized according to the income deciles of the heads of households, and eight sectors were systematically drawn in each decile. Subsequently, a step was taken to reduce the coefficient of variation of the households in each sector, by dividing the sectors with the largest number of households (> 500) and grouping those with the lowest number (< 150), which resulted in 83 sectors, composed of a total of 22,846 households. At baseline, 1,911 older adults (≥ 60 years old) were identified and considered eligible.

Data collection was performed with a standardized questionnaire, applied as a face-to-face interviews at the participant’s residence, which offered registration data necessary for geolocation, containing the participant’s identification code (ID), name, telephone, street, residential number, residential postal code (ZIP code), and neighborhood.

It had three waves of assessment—baseline (2009–2010), follow-up after five years (2013–2014), and follow-up after 10 years (2017–2019)—with the first wave involving 1,705 respondents. However, two duplicate participants and one with incompatible age took the sample to 1,702, keeping the response rate at 89.2%. The second wave reached 1,197 participants, and from the third, it became an open cohort with 1,335 participants, of which 743 were follow-up interviews, 105 were older adults from the EpiFloripa Adult sample, and 487 were new recruits^23^. Further methodological details can be found in previous studies^20,23,24^.

**Figure 1:**
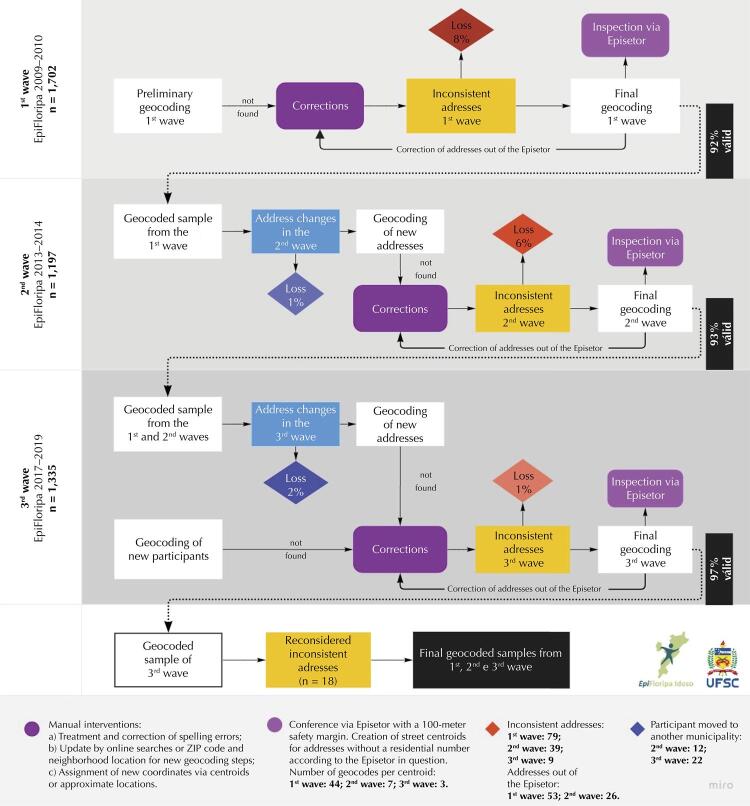
Geocoding processes applied in three monitoring waves in Florianópolis. EpiFloripa Ageing Cohort Study, 2009–2019.

The geocoding procedure followed several steps in this study, with three main strategies: a) address standardization; b) manual correction; and c) coordinate assignment and conference (Figure 1). The recurrence of incomplete address records or those with formatting incompatible with the geocoding program required standardization and normalization in a format suitable for import. For a low-cost procedure that does not require trained staff, we opted for the free Google Earth Pro software. The same software was chosen for the availability of qualified researchers and for its ability to quickly and automatically process the coordinates corresponding to the addresses^9^, suggesting corrections for invalid addresses.

To assess the coverage (proportion of successfully geocoded addresses) and positional accuracy of the participants’ households (how close the geocoded coordinates correspond to the true coordinates)^11^, a preliminary geocoding of the baseline was generated (EpiFloripa Idoso, 2009-2010). It highlighted the need to correct the addresses, preparing them for a definitive importation.

Strategies used to deal with incomplete addresses are among the main determinants of geocoding positional error^11^. Thus, addresses that were not found were verified on a case-by-case basis (Figure 1). The correction process involved processing the database (Microsoft Excel 2013) and updating the addresses via consultation of additional reported data. Searches on mapping sites (Google Maps, Google Street View) and municipal road system data (http://geo.pmf.sc.gov.br) favored the manual geocoding of the coordinates of addresses that were not found.

Due to the change in the number of census tracts by the IBGE between the 2000 and 2010 censuses, we chose to group sectors with similar mean income *per capita* characteristics, to guarantee a minimum number of older adults in each location. Thus, the study created what was called an Episector: a grouping of adjacent census tracts with similar characteristics, considering their geographical location and corresponding income decile^15^. The same grouping was used as a mechanism to verify geocoding.

To avoid sample loss, participants recruited in the first wave who lived outside the boundary of the selected Episector were reconsidered based on a safety margin defined by the average size of a block (100 meters from the surroundings of the Episectors). Thus, data from individuals living at the edges of the census tract and who are within its zone of influence were safeguarded. For the participants in the three waves of the study, the location outside the tolerance margin of the Episector was disregarded as an error factor, favoring longitudinal studies.

In similar studies, inaccessible addresses were solved by generating a “midpoint of the street segment,” deriving a centroid^6,25^. Therefore, for participants without records related to the residential number and without possibility of contact, the latitude/longitude coordinates of the centroids of the informed street were assigned. In extensive streets, the numbering of houses within the Episector in question was sought.

The same spatialization criteria were followed for the second and third waves of the study. Participants who changed addresses between the waves of research had their new home address checked and formatted for a new geocoding.

Participants with valid addresses were analyzed regarding 19 variables derived from the EpiFloripa Ageing, which encompass blocks of the questionnaire with sociodemographic data, data of perception of the environment, and health conditions along three waves of follow-up. The information collection method has been described in previous studies^20,23,24^. The data were compared according to the total samples, to identify the effect of georeferencing losses on the sample data of the three waves. The significance (95%) of the difference between the values for the total sample and the geocoded sample was calculated from a Z test for proportions.

**Figure 2:**
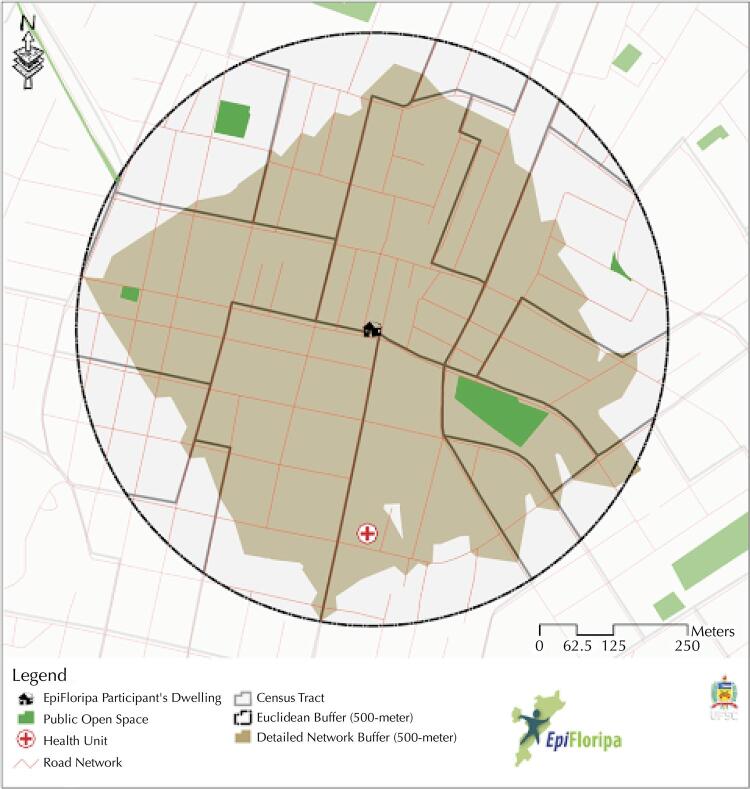
Comparison between three types of neighborhood definition, census tract, Euclidean buffer, and detailed network buffer. EpiFloripa Ageing Cohort Study, 2009–2019.

Neighborhood definitions were adopted according to three different units of spatial analysis (Figure 2). From the database of streets in the municipality (Florianópolis City Hall – PMF – 2012), Euclidean (circular) and network (detailed) buffers were generated, which were then compared with the area pre-delimited by the traditional analysis unit, the census sector. The dimension adopted for the buffer (500 meters) follows previous studies based on a distance that allows an active displacement^26^ and on the average gait speed according to age group^27^, representing 10 minutes of walking from home.

By investigating the differences regarding the three neighborhood definitions, seven environmental variables were calculated for each spatial unit of analysis. For the samples geocoded in the three waves, the variables area (km^2^), mean income *per capita* (census tracts^28^), residential density (housing per hectare), mixed land use (entropy), street connectivity (three intersections or more), and health units and public open spaces counts were calculated^29^. When using buffered census data, the sectors and the portion comprised by them were considered, weighting the values according to the area of each census tract contained therein. To perform the calculations, scripts were created in the QGIS Graphical Modeler, combining different analyses into a single process and containing the analysis unit as a calculation parameter.

Medians and standard deviations were calculated for the variables income, residential density, and entropy. Finally, Pearson’s correlations between the representations by network buffer, circular buffer, and census tracts indicated the relationship between the spatial units for the same three variables. Scatter plots were used to represent the relationship between network buffers and census tract values for the three variables, showing how the different representations resulted in similar or different values.

## RESULTS

Figure 1 shows the quantity of successful geocoding and the description of the specificities of the addresses during the three waves of data collection. The baseline data of the EpiFloripa Aging (2009–2010) required the highest percentage of adjustment (17% of the records were incomplete, n_w1_ = 301) and generated a higher number of losses than the other waves (n_w1_ = 132). Error correction and verification from the expanded limit of the Episector (census tract) identified addresses outside it, inconsistent, and without numerical data (geocoded by the centroid of the street). The second wave of the study (2013-2014) had 77 losses, and the third (2017–2019) had 31, most of which were due to the move to another municipality (n_w3_ = 22). Finally, reconsidering participants from the three study waves with residential locations outside the expanded limit of their respective Episector avoided 18 losses (Figure 1).

### Comparison between Total Sample and Geocoded Sample

Table 1 shows the percentage distribution and p-value according to sociodemographic data, environment and health condition perception of the total sample compared with the georeferenced sample, for the three follow-up waves.

**Table 1:** Older adults’ sociodemographic variables, environmental perception, and health conditions over three follow-ups in Florianópolis according to the total and georeferenced samples. EpiFloripa Aging Cohort Study, 2009–2019.

Variable	1^st^ wave (2009–2010)			2^nd^ wave (2013–2014)			3^rd^ wave (2017–2019)		
		
	Total sample (n = 1,702)	Georeferenced (n = 1,570)	p-value	Total sample (n = 1,197)	Georeferenced (n = 1,120)	p-value	Total sample (n = 1,335)	Georeferenced (n = 1,304)	p-value
	n (%)	n (%)		n (%)	n (%)		n (%)	n (%)	
Sociodemographic									
Gender									
Male	614 (36.1)	561 (35.7)	0.5948	419 (35.0)	386 (34.5)	0.5987	510 (38.2)	499 (38.3)	0.4801
Female	1,088 (63.9)	1,009 (64.3)	0.4052	778 (65.0)	734 (65.5)	0.4013	825 (61.8)	804 (61.7)	0.5199
Age (years)									
≤ 69	848 (49.8)	789 (50.3)	0.3859	412 (34.4)	392 (35)	0.3821	461 (34.5)	449 (34.5)	0.500
70–79	616 (36.2)	565 (36)	0.5478	509 (42.5)	475 (42.4)	0.5199	554 (41.5)	538 (41.3)	0.5398
≥ 80	238 (14)	215 (13.7)	0.5987	276 (23.1)	253 (22.6)	0.6141	320 (24)	316 (24.3)	0.4286
Per capita income (R$ minimum wage)^a^									
≤ 1	197 (11.9)	179 (11.7)	0.5714	92 (8.1)	84 (7.9)	0.5714	140 (10.6)	132 (10.3)	0.5987
2–3	435 (26.3)	400 (26.2)	0.5239	327 (28.7)	310 (29.1)	0.4168	367 (27.8)	361 (28)	0.4562
4 –5	314 (19)	287 (18.8)	0.5596	227 (19.9)	205 (19.2)	0.6628	247 (18.7)	244 (19)	0.4207
6–10	382 (23.1)	350 (23)	0.5279	274 (24.0)	255 (23.9)	0.5239	327 (24.8)	316 (24.6)	0.5478
11–40	310 (18.7)	291 (19.1)	0.3859	216 (18.9)	207 (19.4)	0.3783	233 (17.7)	229 (17.8)	0.4721
> 41	17 (1.0)	17 (1.1)	0.3897	5 (0.4)	5 (0.5)	0.3594	5 (0.4)	5 (0.4)	0.500
Schooling^b^ (years)									
≤ 4	753 (44.2)	685 (43.7)	0.6141	523 (43.7)	486 (43.4)	0.5596	472 (35.5)	463 (35.7)	0.4562
5–8	307 (18)	284 (18.1)	0.4721	199 (16.6)	187 (16.7)	0.4761	244 (18.3)	235 (18.1)	0.5517
9–11	241 (14.2)	218 (13.9)	0.5987	180 (15)	168 (15)	0.5	215 (16.2)	210 (16.2)	0.500
≥ 12	401 (23.6)	380 (24.3)	0.3192	295 (24.6)	279 (24.9)	0.4325	399 (30)	390 (30)	0.500
Environmental perception									
Presence of sidewalks	1,276 (74.8)	1,182 (75.3)	0.3707	881 (73.6)	833 (74.4)	0.33	1,101 (82.7)	1,073 (82.5)	0.5557
Presence of flat streets	800 (46.9)	743 (47.3)	0.409	544 (45.4)	506 (45.2)	0.5398	710 (53.3)	689 (53)	0.5596
Traffic conditions (traffic DOES NOT hinder physical activity)	1,030 (60.4)	952 (60.6)	0.4522	680 (56.8)	634 (56.6)	0.5398	850 (63.9)	829 (63.9)	0.5
Presence of crosswalks	1,027 (60.2)	949 (60.4)	0.4522	756 (63.2)	711 (63.5)	0.4404	933 (70.2)	909 (70)	0.5438
Presence of street lighting	1,490 (87.4)	1,386 (88.3)	0.2148	1,044 (87.2)	986 (88)	0.281	1,204 (90.9)	1,175 (90.9)	0.5
Daytime safety	1,273 (74.7)	1,174 (74.8)	0.4707	942 (78.7)	886 (79.1)	0.4052	1,102 (82.9)	1,076 (82.9)	0.5
Nighttime safety	539 (31.6)	499 (31.8)	0.4522	426 (35.6)	399 (35.6)	0.5	580 (45)	564 (44.9)	0.5199
Presence of public spaces (parks, squares, walking paths, and sports courts)	595 (34.9)	554 (35.3)	0.4052	590 (49.3)	549 (49)	0.5557	851 (64.1)	832 (64.1)	0.5
Health condition									
Overweight^c^	862 (52.5)	801 (52.9)	0.409	622 (54.2)	584 (54.2)	0.5	708 (56.3)	691 (56.2)	0.5199
Health perception									
Very good	156 (9.4)	144 (9.4)	0.5	96 (8.7)	93 (8.7)	0.5	136 (10.6)	135 (10.8)	0.4325
Good	691 (41.8)	640 (41.8)	0.5	524 (47.6)	510 (47.6)	0.5	658 (51.4)	642 (51.3)	0.5199
Regular	640 (38.8)	594 (38.8)	0.4761	402 (36.5)	392 (36.6)	0.4801	420 (32.8)	409 (32.7)	0.5199
Poor	125 (7.6)	117 (7.6)	0.5	59 (5.4)	59 (5.5)	0.4562	56 (4.4)	55 (4.4)	0.5
Very poor	40 (2.4)	37 (2.4)	0.5	20 (1.7)	17 (1.6)	0.5753	11 (0.9)	10 (0.8)	0.8212
Diabetes	376 (22.1)	350 (22.3)	0.4443	301 (25.1)	286 (25.5)	0.4129	336 (25.2)	324 (24.9)	0.5714
Hypertension	1,007 (59.1)	935 (59.6)	0.3859	781 (65.2)	736 (65.7)	0.4013	819 (61.3)	802 (61.6)	0.4364
Depressive symptoms	427 (25.1)	393 (25.0)	0.5279	342 (28.6)	316 (28.2)	0.5832	300 (22.5)	292 (22.4)	0.5239
Cognitive deficit^d^	453 (26.7)	400 (25.6)	0.7642	306 (25.9)	282 (25.5)	0.5871	258 (19.5)	254 (19.7)	0.4483
Physical activity^e^	866 (50.9)	801 (51.0)	0.4761	580 (50.5)	565 (50.7)	0.4602	639 (48.6)	625 (48.7)	0.4801

^a^ Per capita income: 1^st^ wave: n = 1,659; 2^nd^ wave: n = 1,147; 3^rd^ wave: n = 1,318.

^b^ Length of schooling: 1^st^ wave: n = 1,694; 2^nd^ wave: n = 1,194; 3^rd^ wave: n = 1,330.

^c^ Overweight: body mass index ≥ 27.0 kg/m^2^.

^d^ Cognitive deficit: categorized based on Almeida (provided in the database).

^e^ Physical activity: sum of time spent on physical activity on commuting and leisure, dichotomized according to recommendations of 150 minutes of moderate to vigorous physical activities per week.

Comparing income and schooling values shows a small bias in the direction of higher incomes and higher education, although these differences are not statistically significant in any of the cases. The geocoded sample showed a reduced proportion of participants with up to 1 minimum wage and an increased proportion of individuals with more than 10 minimum wages. Similarly, the variables related to the environment also show a clear bias towards better conditions of the georeferenced samples compared with the total sample: in both wave 1 and wave 2, the georeferenced sample has more sidewalks, crosswalks, lighting, and safety during the day than the total, whereas only wave 1 has the same effect for the presence of flat streets, traffic conditions, safety at night, and the presence of public spaces. In all cases, however, these differences were not statistically significant.

The same pattern, although less pronounced, occurs for the variables of health perception, depression symptoms, cognitive deficit, and physical activity, which are more favorable in the georeferenced sample than in the total, whereas the reverse is true for overweight, diabetes, and hypertension.

Table 2 presents seven descriptive variables for the three spatial units considered here: census tract, circular buffers, and network buffers. In general, the standard deviations of the two types of buffers are smaller than those of the census tracts. The values of the environmental characteristics for the three units indicate low variability between the neighborhoods along the three lines of study, except for the contextual income, which showed an increase. Attributes such as mixed land use, number of health units, and number of public open spaces maintain lower values over three follow-up waves. The low values, evidenced by the three units of analysis, reveal a lower access to different land uses, and a limited access to health and leisure equipment in the sampled neighborhoods.

**Table 2:** Neighborhood characteristics of older adults’ residence over three follow-up waves in Florianopolis according to geocoded samples. EpiFloripa Ageing Cohort Study, 2009–2019. (nw1 = 1,570; nw2 = 1,120; nw3 = 1,304).

Variable	Census tract	Buffer - 500-meter	

		Circular	Detailed network
1^st^ wave (2009–2010)	Median (SD)	Median (SD)	Median (SD)
Area (km^2^) of the neighborhood definition type	0.14 (0.57)	0.79	0.23 (0.09)
Income (R$): average per capita income (IBGE, 2010)	1,428.11 (944.10)	1,443.49 (826.37)	1,457.80 (899.21)
Residential density^a^: sum of houses per street (IBGE, 2019)	17.41 (52.21)	21.30 (17.27)	29.72 (25.57)
Land use mix (entropy): balance between seven different land uses (IBGE, 2010)	0.12 (0.09)	0.10 (0.06)	0.12 (0.07)
Street connectivity^a^	9.00 (7.61)	42.00 (29.37)	22.00 (18.24)
Number of health units	0.00 (0.31)	0.00 (0.77)	0.00 (0.56)
Number of POS	0.00 (1.09)	1.00 (2.01)	0.00 (1.27)
2^nd^ wave (2013–2014)	Median (SD)	Median (SD)	Median (SD)
Area (km^2^)	0.16 (0.59)	0.79	0.23 (0.09)
Income (R$)	1,447.83 (939.07)	1,469.45 (822.68)	1,471.14 (899.99)
Residential density^a^	17.57 (57.16)	21.79 (17.40)	30.23 (25.92)
Land use mix (entropy)^b^	0.12 (0.10)	0.10 (0.06)	0.12 (0.07)
Street connectivity^c^	10.00 (7.82)	42.00 (30.31)	22.00 (18.89)
Number of health units	0.00 (0.31)	0.00 (0.77)	0.00 (0.56)
Number of POS	0.00 (1.08)	1.00 (1.95)	0.00 (1.23)
3^rd^ wave (2017–2019)	Median (SD)	Median (SD)	Median (SD)
Area (km^2^)	0.16 (0.66)	0.79	0.25 (0.10)
Income (R$)	1,499.71 (955.52)	1,487.45 (821.17)	1,467.73 (884.97)
Residential density^a^	17.57 (44.57)	20.10 (16.86)	29.24 (24.88)
Land use mix (entropy)	0.12 (0.10)	0.10 (0.06)	0.12 (0.08)
Street connectivity^b^	9.00 (8.21)	40.00 (33.07)	23.00 (20.52)
Number of health units	0.00 (0.31)	0.00 (0.79)	0.00 (0.60)
Number of POS	0.00 (0.96)	1.00 (2.12)	0.00 (1.34)

POS: public open space.

Note: the circular buffer size is the same for all cases.

^a^ Number of households by line segment corresponding to street facets. Available at the National Register of Addresses for Statistical Purposes, IBGE, 2019.

^b^ The Entropy formula stems from the sum of the proportions of each land use in a spatial unit, weighted by the Napierian logarithm of these proportions (Shannon, 1948). Values range from 0, in which the entire area has a single use, to 1, in which the uses under analysis are divided equally in a spatial unit (Park et al. [2018]). Seven categories of land use were considered following the classification used in Epifloripa: 1. Residences; 2. Supermarkets, convenience stores/mini markets/grocery stores, farmers’ markets; 3. Stores, bookstores, banks, pharmacies, beauty salons, barbershops; 4. Restaurants, bakeries, snack bars, coffee shops; 5. Health centers, community centers; 6. Parks, public squares, walking lanes, bike lines, sports courts; 7. Gyms and/or clubs.

^c^ Three-way or higher intersection (+3).

Table 3 shows that measures of mixed land use and residential density for circular and network buffers are highly correlated across the three waves, with values ranging from 0.74 to 0.83, whereas the correlation of both types of buffers with census tracts is much lower (0.37 to 0.54). For the income variable, all measures in all spatial units are highly correlated, ranging from 0.85 to 0.97.

**Table 3:** Pearson’s correlation between spatial units regarding income (average per capita income in BLR), residential density (dwellings per hectare), and objective entropy according to geocoded samples. EpiFloripa Ageing Cohort Study, 2009–2019. (nw1 = 1,570; nw2 = 1,120; nw3 = 1,304).

Variable	Network buffer 500 m	Circular buffer 500 m
1^st^ wave (2009–2010)		
Objective entropy		
Network buffer 500 m	-	
Circular buffer 500 m	0.83*	-
Census tract	0.50*	0.54*
Residential density		
Network buffer 500 m	-	
Circular buffer 500 m	0.78*	-
Census tract	0.39*	0.39*
Income		
Network buffer 500 m	-	
Circular buffer 500 m	0.97*	-
Census tract	0.90*	0.86*
2^nd^ wave (2013–2014)		
Objective entropy		
Network buffer 500 m	-	
Circular buffer 500 m	0.82*	-
Census tract	0.51*	0.55*
Residential density		
Network buffer 500 m	-	
Circular buffer 500 m	0.78*	-
Census tract	0.37*	0.37*
Income		
Network buffer 500 m	-	
Circular buffer 500 m	0.97*	-
Census tract	0.89*	0.85*
3^rd^ wave (2017–2019)		
Objective entropy		
Network buffer 500 m	-	
Circular buffer 500 m	0.74*	-
Census tract	0.46*	0.56*
Residential density		
Network buffer 500 m	-	
Circular buffer 500 m	0.81*	-
Census tract	0.41*	0.42*
Income		
Network buffer 500 m	-	
Circular buffer 500 m	0.97*	-
Census tract	0.88*	0.85*

*p < 0.05.

## DISCUSSION

The geocoding of data from the EpiFloripa Ageing Cohort Study with Google Earth Pro had a high proportion of matches, despite the difficulties related to inconsistencies in the addresses. Among the residential data of the three study waves, only 6% (n_w1,w2,w3_ = 240) were considered losses, and 1% (n_w1,w2,w3_ = 44) received coordinates corresponding to the centroid of their respective street, which led to the absence of statistically significant difference between the total sample and the georeferenced sample (Table 1).

Although the coordinate assignment rate approached 100%, a significant part of the losses involved addresses that were not found (n_w1_ = 79; n_w2_ = 39; n_w3_ = 9). This fact is partially justified by the physical-geographical characteristics of the municipality and its historical occupation process. The previous rural structuring and naval flows led to the formation of a disjointed and fragmentary urban fabric, with the presence of fishbone traces, varied easements, and disconnected and peripheral neighborhoods^30^. In addition, the slight difference in the proportion of income groups indicates possible problems related to geocoding populations of neighborhoods of lower socioeconomic status (Table 1).

In the insular portion, low-income settlements are located on hillsides and in areas with little accessibility^30^. The irregularity and urban exclusion impose inequalities in the municipal registry, implying difficulties in georeferencing. This problem is not unique to the research: another Brazilian study^19^ revealed weaknesses in the geocoding of less urbanized sectors, neighborhoods of lower socioeconomic level, and recent settlements, with irregular completeness and precision, which may impact public health and education actions precisely in areas that need them most.

Another factor that may justify the volume of losses is the small number of interviewers in the field in the first wave of the study, their turnover, and the need for replacement in the second wave^9^. These factors generated limitations in the accuracy and rigor of the procedure for registering the participants’ home addresses. Additionally, 53 addresses were located outside the Episector, excluding participants of the three waves (n_w1,w2,w3_ = 18). These results reinforce the need for epidemiological studies to include in their planning training on ways to obtain address data with greater quality or accuracy, or to use other forms of geolocation, such as mobile devices for real-time location (e.g., mobile phones, portable GPS, among others). This can ensure higher quality of the georeferenced data.

Regarding the possibility of introducing a bias with the losses imposed by geocoding, the p-values in Table 1 indicate that, for all considered variables, and for the three waves, the total sample and the georeferenced sample showed no statistically significant difference. That is, the losses in the georeferencing of the three study waves did not affect their representativeness compared with the total sample. Despite this, all variables of built environment perception showed a slight increase in the georeferenced sample. Considering that higher values in these characteristics indicate areas with higher quality (greater presence of sidewalks, greater safety during the day and at night, etc.), this suggests that the geocoding process may have inserted a small (and statistically insignificant) distortion of including a higher proportion of participants with better levels of income, education, and living conditions. The proportions of income groups confirm this impression, reinforcing what was previously commented on the greater amount of losses in areas with more socioeconomic problems.

On the other hand, although the process caused sample losses, geocoding allowed the adoption of buffers, evidencing their statistical difference compared with measures calculated by census tracts, and highlighting flaws in describing the spatial attributes calculated on this territorial unit. The artificial spatial standardization of the census tract creates units of different dimensions and aggregation levels, which generated spatial measures with high variation (larger standard deviations) compared with buffer-based measures, especially for measures such as area, income, residential density, and mixed land use (Table 2). Pearson’s correlation coefficients showed little correspondence between the measures calculated by the different spatial units during the three study waves, except for the income measure, calculated with data at the census tract level (Table 3). This was probably due to limitations in the data source causing aggregation in buffers to use data from the census tracts themselves. The results point to the influence of the use of census tracts on findings of spatial epidemiological analyses^6^, suggesting that adopting buffers can help manage their limitations, representing a more effective aggregation unit of environmental data^7,13^.

Due to these problems, we recommend that household-based surveys standardize records, expanding the detailing of location information^9^. The use of specific software and programming for normalization and search of the input addresses could have reduced the time spent updating the problematic addresses. Therefore, future studies may employ different geocoding methods, comprising address verification algorithms^16^, precision measurements of geocoded locations, and positional error assessments. Similarly, we recognize the need for a team familiar with geocoding and data manipulation software.

Finally, the low quality of municipal records in peripheral areas highlights a problem that impacts knowledge about urban reality and limits the creation of evidence-based public policies aimed at the most vulnerable populations. Therefore, the need to improve municipal registries is highlighted, expanding the detailing of location information that serves as input for geocoding.
