# Supplementary material for: Geocoding processes in cohort studies: methods applied in the EpiFloripa Aging
Source: Rev Saude Publica. 2023 Nov 8;57:88. doi: 10.11606/s1518-8787.2023057004976 (PMC10681526; doi:10.11606/s1518-8787.2023057004976)
Supplement: Translation

# Translation: Processos de geocodificação em estudos de coorte: métodos aplicados no EpiFloripa Idoso

**Authors:** Catharina Cavasin Salvador, Adalberto Aparecido dos Santos Lopes, Danilo Resendes, Fernanda Faccio Demarco, Marcelo Dutra Della Justina, Renato Tibiriçá de Saboya, Cassiano Ricardo Rech, Eleonora d’Orsi

**Affiliations:** I Brasil Universidade Estadual de Londrina. Programa Associado de Pós-Graduação em Arquitetura e Urbanismo. Londrina, PR, Brasil; II Brasil Universidade Federal de Santa Catarina. Programa de Pós-Graduação em Arquitetura e Urbanismo. Florianópolis, SC, Brasil; III Brasil Universidade Federal de Minas Gerais. Observatório de Saúde Urbana. Belo Horizonte, MG, Brasil; IV Brasil Universidade Federal de Santa Catarina. Programa de Pós-Graduação em Educação Física. Florianópolis, SC, Brasil; V Brasil Universidade Federal de Santa Catarina. Centro Tecnológico. Departamento de Arquitetura e Urbanismo. Florianópolis, SC, Brasil; VI Brasil Universidade Federal de Santa Catarina. Centro de Desportos. Departamento de Educação Física. Florianópolis, SC, Brasil; VII Brasil Universidade Federal de Santa Catarina. Centro de Ciências da Saúde. Departamento de Saúde Pública. Florianópolis, SC, Brasil

**Keywords:** Saúde do Idoso, Meio Ambiente e Saúde Pública, Inquéritos Epidemiológicos, Mapeamento Geográfico, Sistemas de Informação Geográfica, Análise Espacial

## Abstract

**OBJETIVO:**

Descrever o processo e as implicações epidemiológicas do georreferenciamento nas amostras do EpiFloripa Idoso (2009–2019).

**MÉTODO:**

O estudo de coorte EpiFloripa Idoso buscou investigar e acompanhar as condições de vida e saúde da população idosa (≥ 60) de Florianópolis em três ondas de estudo (2009/2010, 2013/2014, 2017/2019). Com uma ferramenta de geocodificação automática, os endereços residenciais foram espacializados, permitindo a investigação do efeito das perdas amostrais do georreferenciamento em relação a 19 variáveis, avaliadas nas três ondas. A influência de diferentes definições de vizinhança (setores censitários, buffers euclidianos e buffers pela rede de ruas) foi examinada nos resultados de sete variáveis: área, renda, densidade residencial, uso misto do solo, conectividade, contagem de unidades de saúde, e contagem de espaços livres públicos. Coeficientes de correlação de Pearson foram calculados para avaliar as diferenças entre as definições de vizinhança de acordo com três variáveis: renda contextual, densidade residencial e diversidade de uso do solo.

**RESULTADO:**

As perdas impostas pela geocodificação (6%, n = 240) não ocasionaram diferença estatística significativa entre a amostra total e a georreferenciada. A análise das variáveis do estudo sugere que o processo de geocodificação pode ter incluído uma maior proporção de participantes com melhor nível de renda, escolaridade e condições de vida. Os coeficientes de correlação evidenciaram pouca correspondência entre medidas calculadas pelas três definições de vizinhança (r = 0,37–0,54). A diferença estatística entre as variáveis calculadas por buffers e setores censitários ressalta limitações no uso destes na descrição dos atributos geoespaciais.

**CONCLUSÃO:**

Apesar dos desafios relacionados à geocodificação, como inconsistências nos endereços, adequados mecanismos de correção e verificação propiciaram elevada taxa de atribuição de coordenadas geográficas. Os achados sugerem que a adoção de buffers, favorecida pela geocodificação, representa uma potencialidade para análises epidemiológicas espaciais ao aprimorar a representação dos atributos do ambiente e a compreensão dos desfechos de saúde.

## INTRODUÇÃO

Com o aumento da população mundial urbana, um número crescente de investigações busca compreender as relações entre os ambientes urbanizados e desfechos de saúde^1^. Planejar e gerir as cidades de forma eficiente possui potencial de promover a saúde e o bem-estar, além de reduzir a incidência de doenças crônicas não transmissíveis^2,3^, com um efeito duradouro^4^. Os Sistemas de Informações Geográficas (SIG) são um conjunto de tecnologias que permitem integrar, em um mesmo ambiente, variáveis sobre diferentes aspectos da realidade e em diferentes escalas de agregação^5,6^. Modelos geográficos baseados em SIG sustentam na análise de disparidades de saúde conceitos como contexto de vizinhança, disponibilidade de serviços de saúde, prática de atividade física e acessibilidade a destinos cotidianos^7^, capazes de contribuir para trabalhos sobre saúde e qualidade de vida nas cidades.

Os avanços em SIG nas últimas duas décadas aumentaram a especificidade com a qual o ambiente da vizinhança de um indivíduo pode ser espacialmente definido^8^. Análises em SIG na área de Saúde Coletiva são geralmente baseadas na localização residencial de um indivíduo, que pode ser definida em vários níveis de resolução geográfica, como: a) limites administrativos (como bairros, municípios ou outras regionalizações); b) setores censitários (unidade territorial definida a cada censo pelo Instituto Brasileiro de Geografia e Estatística, IBGE, para controlar a coleta de dados populacionais); e, c) latitude e longitude de um endereço residencial. Para limites administrativos e setores censitários, não é necessário converter o endereço em coordenada, no entanto deve-se observar a correspondência do endereço com o limite territorial em estudo. Por outro lado, o último exige um processo de conversão de endereços textuais em coordenadas geográficas, conhecido como geocodificação^6,11^.

A importância da geocodificação para análise de dados de saúde tem sido evidenciada por pesquisas nacionais^12^.

A geocodificação permite a adoção de *buffers,* uma zona em torno do endereço residencial de um indivíduo (ponto) que estabelece uma área limite, definida por uma distância máxima especificada, onde são agregados dados espaciais de interesse. Os *buffers* definem e caracterizam a vizinhança com precisão, ajudando a gerenciar limitações dos setores censitários e o problema da unidade de área modificável^7^. Apesar da importância da escala para agregar as variáveis do ambiente, poucas pesquisas examinaram a influência de diferentes definições de vizinhança em resultados de análises^13^. A partir disso, percebe-se que os resultados dos atributos objetivos do ambiente urbano adquiridos com cada tipo de resolução geográfica podem ser diferentes, superestimando ou subestimando a real exposição que os participantes de um estudo epidemiológico têm aos atributos de interesse da investigação.

Embora a agilidade na espacialização de um grande volume de locais seja uma vantagem da geocodificação, o processo de conversão potencializa o risco de erros de posição e classificação. Trabalhos anteriores reportaram taxas de geocodificação variáveis e perdas causadas por endereços problemáticos e com má qualidade dos registros^14^. Erros podem gerar descrições incorretas das variáveis do ambiente construído, conclusões distorcidas sobre a associação entre variáveis dependentes e independentes, e decisões de saúde pública inadequadas^11^. Estudos internacionais utilizam o ArcGIS(r)/ArcView(r), um software licenciado para a geocodificação^6^, porém apontam riscos de localização incorreta^6^ e erros quando aplicados em outros países^19^. Outras pesquisas contratam empresas comerciais com profissionais treinados, softwares próprios e correções espaciais contínuas^18^. Portanto, para minimizar as despesas de geocodificação interna, dados locacionais de alta qualidade são fundamentais.

O estudo de coorte EpiFloripa Idoso, realizado em Florianópolis, Santa Catarina, buscou investigar e acompanhar as condições de vida e saúde da população idosa (60 anos ou mais) residente na zona urbana do município^20^. As publicações provenientes deste projeto têm utilizado, até então, os setores censitários como unidade espacial de análise e representação das vizinhanças dos participantes^21,22^. Com a geocodificação dos domicílios, novos estudos podem ser desenvolvidos, aplicando unidades de análise mais específicas ao ambiente urbano que efetivamente possa ser acessado dentro de um determinado intervalo de tempo. Contudo, esse processo impõe diversos desafios tecnológicos e operacionais que precisam ser enfrentados para assegurar confiabilidade e precisão aos resultados.

Assim, o objetivo deste estudo é descrever o processo e as implicações epidemiológicas da geocodificação das residências dos participantes da amostra do EpiFloripa Ageing Cohort Study (2009–2019). Para estas últimas, mais especificamente, fazemos: a) uma comparação entre dados sociodemográficos, de percepção do ambiente e de condição de saúde obtidos para a amostra total e aquela proporção que foi geocodificada, em busca de possíveis distorções; e b) uma comparação do desempenho de três definições de vizinhança possíveis a partir da geocodificação (setores censitários, buffers euclidianos e buffers pela rede de ruas) para algumas variáveis relevantes, como renda, densidade residencial, uso misto do solo e conectividade.

## MÉTODOS

O projeto EpiFloripa Idoso é um estudo de coorte de base populacional desenvolvido pela Universidade Federal de Santa Catarina^23^. O contexto espacial do estudo envolve todo o município de Florianópolis (SC), com 421.240 habitantes, e 11,4% da população composta por pessoas acima de 60 anos de idade^19^. O processo de seleção da amostra foi realizado por conglomerados, nos quais as unidades de primeiro estágio foram os setores censitários e as do segundo estágio foram os próprios domicílios. Inicialmente foram organizados, em 2009, os 420 setores censitários urbanos do município conforme os decis de renda dos chefes dos domicílios, e sorteados sistematicamente oito setores em cada decil. Posteriormente, foi realizada uma etapa para redução do coeficiente de variação dos domicílios de cada setor, por meio da divisão dos setores com maior número de domicílios (> 500) e agrupamento daqueles com menor número (< 150), o que resultou em 83 setores, compostos por um total de 22.846 domicílios. Na linha de base, 1.911 idosos (≥ 60 anos) foram identificados e considerados elegíveis.

A coleta de dados foi realizada por meio de um questionário padronizado, aplicado na forma de entrevista face-a-face na residência do participante, o qual ofereceu dados cadastrais necessários para a geolocalização, contendo o código de identificação do participante (ID), nome, telefone, logradouro, número residencial, código postal residencial (CEP) e bairro.

Com três ondas de avaliação – linha de base (2009–2010), acompanhamento após cinco anos (2013–2014), e acompanhamento após 10 anos (2017–2019) –, a primeira onda envolveu 1.705 entrevistados. No entanto, dois participantes duplicados e um com idade incompatível levaram a amostra para 1.702, mantendo a taxa de resposta em 89,2%. A segunda onda obteve 1.197 participantes, e, a partir da terceira, tornou-se uma coorte aberta, com 1.335 participantes, sendo 743 entrevistas de acompanhamento, 105 idosos oriundos da amostra EpiFloripa Adulto, e 487 novos recrutados^23^. Maiores detalhes metodológicos encontram-se em estudos prévios^20,23,24^.

O procedimento de geocodificação seguiu neste estudo várias etapas, com três estratégias principais: a) padronização dos endereços; b) correção manual; e c) atribuição de coordenadas e conferência (Figura 1). A recorrência de registros de endereço incompletos ou com formatação incompatível com o programa de geocodificação exigiu sua padronização e normalização em um formato adequado para importação. Para um procedimento de baixo custo que dispensa equipe treinada, optou-se pelo software gratuito Google Earth Pro. O mesmo software foi escolhido pela disponibilidade de pesquisadores habilitados e por sua capacidade de processar as coordenadas correspondentes aos endereços de forma rápida e automatizada^9^, sugerindo correções para endereços inválidos.

**Figura 1:** Processo de geocodificação e perdas das três ondas de acompanhamento em Florianópolis. Estudo de coorte EpiFloripa Idoso, 2009–2019.

Para avaliar a cobertura (proporção de endereços geocodificados com sucesso) e precisão posicional dos domicílios dos participantes (quão próximas as coordenadas geocodificadas correspondem às coordenadas verdadeiras)^11^, foi gerada uma geocodificação preliminar da linha de base (EpiFloripa Idoso, 2009-2010). Ela evidenciou a necessidade de correção dos endereços, preparando-os para uma importação definitiva.

Estratégias empregadas para lidar com endereços incompletos estão entre as principais determinantes de erro posicional de geocodificação^11^. Assim, a checagem dos endereços não encontrados foi feita caso a caso (Figura 1). O processo de correção envolveu o tratamento da base de dados (Microsoft Excel 2013) e a atualização dos endereços via consulta de dados adicionais relatados. Pesquisas em sites de mapeamento (Google Maps, Google Street View) e em dados municipais de sistema viário (http://geo.pmf.sc.gov.br) favoreceram a geocodificação manual das coordenadas de endereços não encontrados.

Devido à mudança no número de setores censitários pelo IBGE entre os censos de 2000 e 2010, optou-se pelo agrupamento de setores com características semelhantes de renda média per capita, visando garantir um número mínimo de idosos em cada local. Desta forma, o estudo criou o que foi chamado de Episetor: um agrupamento de setores censitários adjacentes com características similares, considerando sua localização geográfica e decil de renda correspondente^15^. O mesmo agrupamento foi utilizado como mecanismo de verificação da geocodificação.

Para evitar a perda amostral, participantes recrutados na primeira onda que residiam fora do limite do Episetor sorteado foram reconsiderados com base em uma margem de segurança definida pelo tamanho médio de uma quadra (100 metros do entorno dos Episetores). Dessa forma, dados de indivíduos residentes nas bordas do setor censitário e que se encontram dentro de sua zona de influência foram resguardados. Em casos de participantes das três ondas do estudo, foi desconsiderada a localização fora da margem de tolerância do Episetor como um fator de erro, favorecendo estudos longitudinais.

Em estudos semelhantes, endereços inacessíveis foram solucionados a partir da geração de um “ponto médio do segmento de rua”, derivando um centroide^6,25^. Logo, para participantes sem registros relativos ao número residencial e sem possibilidade de contato, foram atribuídas as coordenadas de latitude/longitude dos centroides dos logradouros informados. Em logradouros extensos, buscou-se a numeração de casas dentro do Episetor em questão.

Para as segunda e terceira ondas do estudo, foram seguidos os mesmos critérios de espacialização. Participantes que mudaram de endereço entre as ondas de pesquisa tiveram seu novo endereço residencial conferido e formatado para uma nova geocodificação.

Participantes com endereços válidos foram analisados em relação a 19 variáveis derivadas do EpiFloripa Idoso, as quais englobam blocos do questionário com dados sociodemográficos, dados de percepção do ambiente, e condições de saúde ao longo de três ondas de acompanhamento. O método de coleta dessas informações está descrito em estudos prévios^20,23,24^. Os dados foram comparados de acordo com as amostras totais, visando identificar o efeito das perdas de georreferenciamento nos dados amostrais das três ondas. A significância (95%) da diferença entre os valores para a amostra total e a amostra geocodificada foi calculada a partir de um teste Z para proporções.

Foram adotadas definições de vizinhança de acordo com três diferentes unidades de análise espacial (Figura 2). A partir da base de dados de logradouros do município (Prefeitura Municipal de Florianópolis – PMF – 2012), foram gerados buffers euclidianos (circulares) e de rede (detalhados), que em seguida foram comparados com a área pré-delimitada pela unidade de análise tradicional, o setor censitário. A dimensão adotada para o buffer (500 metros) segue estudos anteriores pautados em uma distância que permita um deslocamento ativo^26^ e na velocidade média de marcha de acordo com a faixa etária^27^, representando 10 minutos de caminhada a partir do domicílio.

**Figura 2:** Comparação entre três tipos diferentes de definição de bairro: setor censitário, buffer euclidiano (circular) e buffer de rede. EpiFloripa Ageing Cohort Study, 2009–2019.

Ao investigar as diferenças em relação às três definições de vizinhança, foram gerados cálculos de sete variáveis ambientais calculadas por cada unidade espacial de análise. Para as amostras geocodificadas nas três ondas, foram calculadas as variáveis área (km^2^), renda *per capita* média (setores censitários^28^), densidade residencial (moradias por hectare), uso misto do solo (Entropia), conectividade das ruas (três intersecções ou mais), e contagem de unidades de saúde e de espaços livres públicos^29^. Ao utilizar dados censitários em buffer, considerou-se os setores e a porção compreendida por ele, ponderando os valores de acordo com a área de cada setor censitário nele contida. Para executar os cálculos, foram criados *scripts* no Modelador Gráfico do QGIS, combinando diferentes análises em um único processo e contendo a unidade de análise como parâmetro para os cálculos.

Medianas e os desvios padrão foram calculados para as variáveis renda, densidade residencial e entropia. Por fim, correlações de Pearson entre as representações por buffer de rede, buffer circular e setores censitários indicaram a relação entre as unidades espaciais para as mesmas três variáveis. Gráficos de dispersão foram utilizados para representar a relação entre buffers de rede e valores de setores censitários para as três variáveis, evidenciando de que forma as diferentes representações resultaram em valores semelhantes ou diferentes.

## RESULTADOS

A Figura 1 apresenta o quantitativo de geocodificações bem-sucedidas e descrição das especificidades dos endereços durante as três ondas de coleta de dados. Os dados da linha de base do EpiFloripa Idoso (2009–2010) exigiram a maior porcentagem de ajuste (17% dos registros estavam incompletos, n_w1_ = 301) e geraram um maior número de perdas em relação às demais ondas (n_w1_ = 132). A correção dos erros e sua verificação a partir do limite expandido do Episetor (setor censitário) identificaram endereços fora dele, inconsistentes e sem dados numéricos (geocodificados pelo centroide do logradouro). A segunda onda do estudo (2013-2014) obteve 77 perdas, e a terceira (2017–2019) obteve 31, sendo a maior parte devido à mudança para outro município (n_w3_ = 22). Por fim, a reconsideração de participantes das três ondas do estudo com localizações residenciais fora do limite expandido de seu respectivo Episetor evitou 18 perdas (Figura 1).

### Comparação entre a Amostra Total e a Amostra Geocodificada

A Tabela 1 expõe a distribuição percentual e o p-valor segundo dados sociodemográficos, de percepção do ambiente, e de condições de saúde da amostra total em comparação com a amostra georreferenciada, para as três ondas de acompanhamento.

**Tabela 1:** Variável sociodemográfica, percepção do ambiente e condição de saúde de idosos ao longo de três ondas de acompanhamento em Florianópolis, de acordo com as amostras totais e georreferenciadas. Estudo de coorte EpiFloripa Idoso, 2009–2019.

Variável	1ª onda (2009–2010)			2ª onda (2013–2014)			3ª onda (2017–2019)		
		
	Amostra total (n = 1.702)	Georreferenciada (n = 1.570)	Valor de p	Amostra total (n = 1.197)	Georreferenciada (n = 1.120)	Valor de p	Amostra total (n = 1.335)	Georreferenciada (n = 1.304)	Valor de p
					
	n (%)	n (%)		n (%)	n (%)		n (%)	n (%)	
Sociodemográfica									
Sexo									
Masculino	614 (36,1)	561 (35,7)	0,5948	419 (35,0)	386 (34,5)	0,5987	510 (38,2)	499 (38,3)	0,4801
Feminino	1.088 (63,9)	1.009 (64,3)	0,4052	778 (65,0)	734 (65,5)	0,4013	825 (61,8)	804 (61,7)	0,5199
Idade (anos)									
≤ 69	848 (49,8)	789 (50,3)	0,3859	412 (34,4)	392 (35)	0,3821	461 (34,5)	449 (34,5)	0,5
70–79	616 (36,2)	565 (36)	0,5478	509 (42,5)	475 (42,4)	0,5199	554 (41,5)	538 (41,3)	0,5398
≥ 80	238 (14)	215 (13,7)	0,5987	276 (23,1)	253 (22,6)	0,6141	320 (24)	316 (24,3)	0,4286
Renda *per capita* (R$ (salário mínimo))^a^									
≤ 1	197 (11,9)	179 (11,7)	0,5714	92 (8,1)	84 (7,9)	0,5714	140 (10,6)	132 (10,3)	0,5987
2–3	435 (26,3)	400 (26,2)	0,5239	327 (28,7)	310 (29,1)	0,4168	367 (27,8)	361 (28)	0,4562
4 –5	314 (19)	287 (18,8)	0,5596	227 (19,9)	205 (19,2)	0,6628	247 (18,7)	244 (19)	0,4207
6–10	382 (23,1)	350 (23)	0,5279	274 (24,0)	255 (23,9)	0,5239	327 (24,8)	316 (24,6)	0,5478
11–40	310 (18,7)	291 (19,1)	0,3859	216 (18,9)	207 (19,4)	0,3783	233 (17,7)	229 (17,8)	0,4721
> 41	17 (1,0)	17 (1,1)	0,3897	5 (0,4)	5 (0,5)	0,3594	5 (0,4)	5 (0,4)	0,5
Escolaridade^b^ (anos)									
≤ 4	753 (44,2)	685 (43,7)	0,6141	523 (43,7)	486 (43,4)	0,5596	472 (35,5)	463 (35,7)	0,4562
5–8	307 (18)	284 (18,1)	0,4721	199 (16,6)	187 (16,7)	0,4761	244 (18,3)	235 (18,1)	0,5517
9–11	241 (14,2)	218 (13,9)	0,5987	180 (15)	168 (15)	0,5	215 (16,2)	210 (16,2)	0,5
≥ 12	401 (23,6)	380 (24,3)	0,3192	295 (24,6)	279 (24,9)	0,4325	399 (30)	390 (30)	0,5
Percepção do ambiente									
Presença de calçadas	1.276 (74,8)	1.182 (75,3)	0,3707	881 (73,6)	833 (74,4)	0,33	1.101 (82,7)	1.073 (82,5)	0,5557
Presença de ruas planas	800 (46,9)	743 (47,3)	0,409	544 (45,4)	506 (45,2)	0,5398	710 (53,3)	689 (53)	0,5596
Condições de trânsito (trânsito NÃO dificulta a prática de atividade física)	1.030 (60,4)	952 (60,6)	0,4522	680 (56,8)	634 (56,6)	0,5398	850 (63,9)	829 (63,9)	0,5
Presença de faixa de pedestres	1.027 (60,2)	949 (60,4)	0,4522	756 (63,2)	711 (63,5)	0,4404	933 (70,2)	909 (70)	0,5438
Presença de iluminação	1.490 (87,4)	1.386 (88,3)	0,2148	1.044 (87,2)	986 (88)	0,281	1.204 (90,9)	1.175 (90,9)	0,5
Segurança durante o dia	1.273 (74,7)	1.174 (74,8)	0,4707	942 (78,7)	886 (79,1)	0,4052	1.102 (82,9)	1.076 (82,9)	0,5
Segurança durante a noite	539 (31,6)	499 (31,8)	0,4522	426 (35,6)	399 (35,6)	0,5	580 (45)	564 (44,9)	0,5199
Presença de espaço público (parques, praças, pista para caminhada e quadras de esportes)	595 (34,9)	554 (35,3)	0,4052	590 (49,3)	549 (49)	0,5557	851 (64,1)	832 (64,1)	0,5
Condição de saúde									
Sobrepeso^c^	862 (52,5)	801 (52,9)	0,409	622 (54,2)	584 (54,2)	0,5	708 (56,3)	691 (56,2)	0,5199
Percepção de saúde									
Muito boa	156 (9,4)	144 (9,4)	0,5	96 (8,7)	93 (8,7)	0,5	136 (10,6)	135 (10,8)	0,4325
Boa	691 (41,8)	640 (41,8)	0,5	524 (47,6)	510 (47,6)	0,5	658 (51,4)	642 (51,3)	0,5199
Regular	640 (38,8)	594 (38,8)	0,4761	402 (36,5)	392 (36,6)	0,4801	420 (32,8)	409 (32,7)	0,5199
Ruim	125 (7,6)	117 (7,6)	0,5	59 (5,4)	59 (5,5)	0,4562	56 (4,4)	55 (4,4)	0,5
Muito ruim	40 (2,4)	37 (2,4)	0,5	20 (1,7)	17 (1,6)	0,5753	11 (0,9)	10 (0,8)	0,8212
Diabetes	376 (22,1)	350 (22,3)	0,4443	301 (25,1)	286 (25,5)	0,4129	336 (25,2)	324 (24,9)	0,5714
Hipertensão	1.007 (59,1)	935 (59,6)	0,3859	781 (65,2)	736 (65,7)	0,4013	819 (61,3)	802 (61,6)	0,4364
Sintomas depressivos	427 (25,1)	393 (25,0)	0,5279	342 (28,6)	316 (28,2)	0,5832	300 (22,5)	292 (22,4)	0,5239
Déficit cognitivo^d^	453 (26,7)	400 (25,6)	0,7642	306 (25,9)	282 (25,5)	0,5871	258 (19,5)	254 (19,7)	0,4483
Atividade física^e^	866 (50,9)	801 (51,0)	0,4761	580 (50,5)	565 (50,7)	0,4602	639 (48,6)	625 (48,7)	0,4801

^a^ Renda *per capita*: 1ª onda: n = 1.659; 2ª onda: n = 1.147; 3ª onda: n = 1.318.

^b^ Escolaridade: 1ª onda: n = 1.694; 2ª onda: n = 1.194; 3ª onda: n = 1.330.

^c^ Sobrepeso: índice de massa corporal ≥ 27,0 kg/m^2^.

^d^ Déficit cognitivo: categorizado a partir de Almeida (categorização fornecida no banco de dados).

^e^ Atividade física: soma do tempo despendido em atividade física no deslocamento e no lazer, dicotomizado de acordo com as recomendações de 150 minutos de atividade física de intensidade moderada à vigorosa por semana.

Uma comparação dos valores de renda e escolaridade mostra que há um pequeno viés na direção de maiores rendas e maiores escolaridades, ainda que essas diferenças não sejam estatisticamente significativas em nenhum dos casos. Cabe notar que a amostra geocodificada apresentou uma redução na proporção de participantes com até 1 salário mínimo e um aumento na proporção de indivíduos com mais de 10 salários mínimos. Da mesma forma, as variáveis relacionadas ao meio ambiente também mostram um claro viés para melhores condições das amostras georreferenciadas em relação à amostra total: tanto na onda 1 quanto na onda 2, a amostra georreferenciada possui mais calçadas, faixas de pedestres, iluminação e segurança durante o dia do que a total, enquanto apenas para a onda 1 o mesmo vale para a presença de ruas planas, as condições de trânsito, a segurança durante a noite e a presença de espaços públicos. Em todos os casos, entretanto, essas diferenças não foram estatisticamente significativas.

O mesmo padrão, ainda que de maneira menos pronunciada, acontece no caso das variáveis de percepção da saúde, sintomas de depressão, déficit cognitivo e de atividade física, que são mais favoráveis na amostra georreferenciada do que na total, enquanto o inverso vale para sobrepeso, diabetes e hipertensão.

A Tabela 2 apresenta sete variáveis descritivas para as três unidades espaciais aqui consideradas: setor censitário, buffers circulares e buffers de rede. Em geral, os desvios padrão dos dois tipos de buffers são menores do que os dos setores censitários. Os valores das características ambientais para as três unidades indicam baixa variabilidade entre as vizinhanças ao longo das três linhas de estudo, exceto para a renda contextual, que demonstrou um aumento. Atributos como uso misto do solo, número de unidades de saúde e números de espaços livres públicos mantêm menores valores ao longo de três ondas de acompanhamento. Os valores baixos, evidenciados pelas três unidades de análise, revelam um menor acesso a diferentes usos do solo, e um acesso limitado a equipamentos de saúde e lazer nas vizinhanças amostradas.

**Tabela 2:** Características da vizinhança de idosos ao longo de três ondas de monitoramento em Florianópolis. de acordo com as amostras geocodificadas. Estudo de coorte EpiFloripa Idoso, 2009–2019. (nw1 = 1.570; nw2 = 1.120; nw3 = 1.304).

Variável	Setor censitário	Buffer - 500 metros	

		Circular	De rede
1^a^ onda (2009–2010)	Mediana (DP)	Mediana (DP)	Mediana (DP)
Área (km^2^) do tipo de definição de vizinhança	0,14 (0,57)	0,79	0,23 (0,09)
Renda (R$): renda média *per capita* (IBGE, 2010)	1.428,11 (944,10)	1.443,49 (826,37)	1.457,80 (899,21)
Densidade residencial^a^: soma de casas por rua (IBGE, 2019)	17,41 (52,21)	21,30 (17,27)	29,72 (25,57)
Combinação de uso do solo (entropia): equilíbrio entre sete usos diferentes do solo (IBGE, 2010)	0,12 (0,09)	0,10 (0,06)	0,12 (0,07)
Conectividade das ruas^a^	9,00 (7,61)	42,00 (29,37)	22,00 (18,24)
Número de unidades de saúde	0,00 (0,31)	0,00 (0,77)	0,00 (0,56)
Número de EPA	0,00 (1,09)	1,00 (2,01)	0,00 (1,27)
2^a^ onda (2013–2014)	Mediana (DP)	Mediana (DP)	Mediana (DP)
Área (km^2^)	0,16 (0,59)	0,79	0,23 (0,09)
Renda (R$)	1.447,83 (939,07)	1.469,45 (822,68)	1.471,14 (899,99)
Densidade residencial^a^	17,57 (57,16)	21,79 (17,40)	30,23 (25,92)
Combinação de uso do solo (entropia)^b^	0,12 (0,10)	0,10 (0,06)	0,12 (0,07)
Conectividade das ruas^c^	10,00 (7,82)	42,00 (30,31)	22,00 (18,89)
Número de unidades de saúde	0,00 (0,31)	0,00 (0,77)	0,00 (0,56)
Número de EPA	0,00 (1,08)	1,00 (1,95)	0,00 (1,23)
3^a^ onda (2017–2019)	Mediana (DP)	Mediana (DP)	Mediana (DP)
Área (km^2^)	0,16 (0,66)	0,79	0,25 (0,10)
Renda (R$)	1.499,71 (955,52)	1.487,45 (821,17)	1.467,73 (884,97)
Densidade residencial^a^	17,57 (44,57)	20,10 (16,86)	29,24 (24,88)
Combinação de uso do solo (entropia)^b^	0,12 (0,10)	0,10 (0,06)	0,12 (0,08)
Conectividade das ruas^c^	9,00 (8,21)	40,00 (33,07)	23,00 (20,52)
Número de unidades de saúde	0,00 (0,31)	0,00 (0,79)	0,00 (0,60)
Número de EPA	0,00 (0,96)	1,00 (2,12)	0,00 (1,34)

EPA: espaços públicos abertos.

Nota: O tamanho do buffer circular é o mesmo para todos os casos.

^a^ Dados de contagem de moradias por segmentos de linhas, correspondentes às faces de logradouros, disponíveis pelo Cadastro Nacional de Endereços para Fins Estatísticos, IBGE, 2019.

^b^ A fórmula da entropia resulta do somatório das proporções de cada uso do solo presente, ponderado pelo logaritmo neperiano dessas proporções (Shannon, 1948). Os valores variam de 0, onde todo o solo possui um único uso, a 1, onde os usos em análise são divididos igualmente na unidade espacial (Park et al. [2018]). Foram consideradas sete categorias de uso do solo, seguindo a classificação utilizada no Epifloripa: 1. Residências; 2. Supermercados, lojas de conveniência / minimercados / mercearias, feiras livres; 3. Lojas, livrarias, bancos, farmácias, salões de beleza, barbearias; 4. Restaurantes, padarias, lanchonetes, cafeterias; 5. Centros de saúde, centros comunitários; 6. Parques, praças públicas, pistas de caminhada, ciclovias, quadras esportivas; 7. Ginásios e/ou clubes.

^c^ Cruzamento de três vias ou mais (+3).

A Tabela 3 mostra que as medidas de uso misto do solo e densidade residencial para buffers circulares e de rede são altamente correlacionadas nas três ondas, com valores variando de 0,74 a 0,83, enquanto a correlação de ambos os tipos de buffers com setores censitários é muito menor (0,37 a 0,54). Para a variável renda, todas as medidas em todas as unidades espaciais são altamente correlacionadas, variando de 0,85 a 0,97.

**Tabela 3:** Correlação de Pearson entre diferentes unidades espaciais de renda (renda média *per capita* em reais), densidade residencial (habitações por hectare) e entropia objetiva, de acordo com amostras geocodificadas. Estudo de coorte EpiFloripa Idoso, 2009–2019. (nw1 = 1.570; nw2 = 1.120; nw3 = 1.304).

Variável	Buffer de rede 500m	Buffer circular 500m
1a onda (2009–2010)		
Entropia objetiva		
Buffer de rede 500m	-	
Buffer circular 500m	0,83*	-
Setor censitário	0,50*	0,54*
Densidade residencial		
Buffer de rede 500 m	-	
Buffer circular 500 m	0,78*	-
Setor censitário	0,39*	0,39*
Renda		
Buffer de rede 500m	-	
Buffer circular 500m	0,97*	-
Setor censitário	0,90*	0,86*
2ª onda (2013–2014)		
Entropia objetiva		
Buffer de rede 500m	-	
Buffer circular 500m	0,82*	-
Setor censitário	0,51*	0,55*
Densidade residencial		
Buffer de rede 500m	-	
Buffer circular 500m	0,78*	-
Setor censitário	0,37*	0,37*
Renda		
Buffer de rede 500m	-	
Buffer circular 500m	0,97*	-
Setor censitário	0,89*	0,85*
3^a^ onda (2017–2019)		
Entropia objetiva		
Buffer de rede 500m	-	
Buffer circular 500m	0,74*	-
Setor censitário	0,46*	0,56*
Densidade residencial		
Buffer de rede 500m	-	
Buffer circular 500m	0,81*	-
Setor censitário	0,41*	0,42*
Renda		
Buffer de rede 500m	-	
Buffer circular 500m	0,97*	-
Setor censitário	0,88*	0,85*

*p < 0.05.

## DISCUSSÃO

A geocodificação dos dados do estudo de coorte EpiFloripa Idoso com o Google Earth Pro teve alta proporção de acertos, apesar das dificuldades relacionadas a inconsistências nos endereços. Dentre os dados residenciais das três ondas de estudo, apenas 6% (n_w1,w2,w3_ = 240) foram considerados perdas, e 1% (n_w1,w2,w3_ = 44) recebeu coordenadas correspondentes ao centroide de seus respectivos logradouros, o que levou à ausência de diferença estatística significativa entre a amostra total e a amostra georreferenciada (Tabela 1).

Embora a taxa de atribuição de coordenadas tenha se aproximado de 100%, uma significativa parte das perdas envolveu endereços não encontrados (n_w1_ = 79; n_w2_ = 39; n_w3_ = 9). Esse fato justifica-se, em parte, pelas características físico-geográficas do município e seu processo histórico de ocupação. A prévia estruturação rural e os fluxos navais levaram à formação de um tecido urbano desarticulado e fragmentário, com a presença de traçados em espinha de peixe, variadas servidões, e bairros desconectados e periféricos^30^. Além disso, a ligeira diferença na proporção dos grupos de renda indica a chance de problemas relativos à geocodificação de populações de bairros de menor nível socioeconômico (Tabela 1).

Na porção insular, assentamentos de baixa renda situam-se em encostas de morros e em áreas com pouca acessibilidade^30^. A irregularidade e a exclusão urbana impõem desigualdades no cadastro municipal, implicando em dificuldades de georreferenciamento. Esse problema não é exclusivo da pesquisa: outro estudo brasileiro^19^ revelou fraquezas na geocodificação de setores menos urbanizados, bairros de menor nível socioeconômico e assentamentos recentes, com completude e precisão irregulares, podendo impactar ações de saúde pública e educação justamente em áreas que mais precisam delas.

Outro fator que pode justificar o volume de perdas é o pequeno número de entrevistadores em campo na primeira onda do estudo, a sua rotatividade e a necessidade de reposição na segunda onda^9^. Esses fatores geraram limitações na precisão e no rigor do procedimento de registro dos endereços residenciais dos participantes. Adicionalmente, 53 endereços tiveram a localização externa ao Episetor, excetuando participantes de três ondas (n_w1,w2,w3_ = 18). Esses resultados reforçam a necessidade de estudos epidemiológicos incluírem em seus planejamentos treinamentos sobre formas de obter dados de endereço com maior qualidade ou precisão, ou utilizarem outras formas de geolocalização, como, por exemplo, dispositivos móveis de localização em tempo real (ex.: celulares, GPS portáteis, entre outros). Isso pode garantir maior qualidade dos dados georreferenciados.

Quanto à possibilidade de introdução de um viés com as perdas impostas pela geocodificação, os valores-p da Tabela 1 indicam que, para todas as variáveis consideradas, e para as três ondas, não houve diferença estatística significativa entre a amostra total e a amostra georreferenciada. Ou seja, as perdas no georreferenciamento das três ondas de estudo não afetaram sua representatividade em relação à amostra total. Apesar disso, todas as variáveis de percepção do ambiente construído mostraram ligeiro aumento na amostra georreferenciada. Considerando que valores maiores nessas características indicam áreas com maior qualidade (maior presença de calçadas, maior segurança durante o dia e à noite etc.), isso sugere que o processo de geocodificação pode ter inserido uma pequena (e estatisticamente insignificante) distorção na direção de incluir maior proporção de participantes com melhores níveis de renda, escolaridade e condições de vida. As proporções dos grupos de renda confirmam essa impressão, reforçando o que foi comentado anteriormente sobre a maior quantidade de perdas em áreas com mais problemas socioeconômicos.

Por outro lado, embora o processo tenha causado perdas amostrais, a geocodificação permitiu a adoção de buffers, evidenciando sua diferença estatística em relação a medidas calculadas por setores censitários, e ressaltando falhas na descrição dos atributos espaciais calculados sobre essa unidade territorial. A padronização espacial artificial do setor censitário cria unidades de diferentes dimensões e níveis de agregação, o que gerou medidas espaciais com alta variação (desvios padrão maiores) em relação às medidas baseadas em buffers, principalmente para medidas como área, renda, densidade residencial e mix de uso do solo (Tabela 2). Os coeficientes de correlação de Pearson evidenciaram pouca correspondência entre as medidas calculadas pelas diferentes unidades espaciais durante as três ondas de estudo, exceto pela medida de renda, calculada com dados a nível de setor censitário (Tabela 3). Isso provavelmente aconteceu porque, devido a limitações na fonte de dados, a agregação em buffers usou dados provenientes dos próprios setores censitários. Os resultados apontam para a influência do uso de setores censitários em achados das análises epidemiológicas espaciais,^6^ sugerindo que a adoção de buffers pode ajudar a gerenciar suas limitações, representando uma unidade de agregação de dados ambientais mais eficaz^7,13^.

Em função dos problemas apontados, recomenda-se que inquéritos de base domiciliar realizem a padronização dos registros, ampliando o detalhamento de informações de localização^9^.O uso de softwares específicos e programação para a normalização e busca dos endereços de entrada poderia ter reduzido o tempo gasto com a atualização dos endereços problemáticos. Portanto, estudos futuros podem empregar diferentes métodos de geocodificação, compreendendo algoritmos de verificação de endereço^16^, medidas de precisão das localizações geocodificadas, e avaliações de erro posicional. Da mesma forma, reconhecemos a necessidade de uma equipe familiarizada com softwares de geocodificação e manipulação de dados.

Por fim, a baixa qualidade dos registros municipais em áreas periféricas destaca um problema que impacta o conhecimento sobre a realidade urbana e limita a criação de políticas públicas baseadas em evidências e voltadas a populações mais vulneráveis. Portanto, destaca-se a necessidade de aprimoramento dos cadastros municipais, ampliando o detalhamento das informações de localização que servem de insumo para a geocodificação.
